# Supplementary material for: Hybrid Models and Biological Model Reduction with PyDSTool
Source: PLoS Comput Biol. 2012 Aug 9;8(8):e1002628. doi: 10.1371/journal.pcbi.1002628 (PMC3415397; doi:10.1371/journal.pcbi.1002628)
Supplement: Text S4 — Complete source code for the PyDSTool package (version 0.88.120504). Includes API documentation and help files linking to web pages. This file is identical to the current public release on Sourceforge.net. (ZIP) [file pcbi.1002628.s004.zip › PyDSTool/html/toc-PyDSTool.Toolbox.data_analysis-module.html]

xml version="1.0" encoding="ascii"?


data\_analysis


# Module data\_analysis

---

## Classes

data\_bins  

## Functions

PCANode

\_adjust\_ix

butter  

colormap

dist\_between\_datasets  
 doPCA  

dump\_progress

filtfilt  
 find\_central\_point  
 find\_closest\_val  
 find\_diameter  
 find\_from\_sorted  
 find\_knees  
 find\_nearby\_annulus  
 find\_nearby\_ball  
 find\_recurrences  
 fitline  
 get\_evec  
 get\_linear\_regression\_residual  
 get\_residual  
 lfilter  
 lfilter\_zi  
 log\_distances  
 log\_distances\_with\_D  
 out\_of\_seq  
 pca\_dim  
 plot\_PCA\_residuals  
 plot\_PCA\_spectrum  
 rectify  

recurrence\_times

second\_diff

whiten  

## Variables

\_PCA\_utils

\_analysis\_utils

\_filter\_utils

\_fit\_utils

\_int\_types

\_metric\_utils

\_num\_types

\_seq\_types

dict\_tol

newaxis

r\_

---

[hide private]
